# *TAC4* controls tiller angle by regulating the endogenous auxin content and distribution in rice

**DOI:** 10.1111/pbi.13440

**Published:** 2020-07-20

**Authors:** Hua Li, Hongying Sun, Jiahuang Jiang, Xianyou Sun, Lubin Tan, Chuanqing Sun

**Affiliations:** ^1^ State Key Laboratory of Plant Physiology and Biochemistry China Agricultural University Beijing China; ^2^ National Center for Evaluation of Agricultural Wild Plants (Rice) Beijing Key Laboratory of Crop Genetic Improvement Laboratory of Crop Heterosis and Utilization MOE Department of Plant Genetics and Breeding China Agricultural University Beijing China

**Keywords:** rice, tiller angle, shoot gravitropism, auxin content and distribution

## Abstract

Tiller angle, an important component of plant architecture, greatly influences the grain yield of rice (*Oryza sativa* L.). Here, we identified *Tiller Angle Control 4* (*TAC4*) as a novel regulator of rice tiller angle. *TAC4* encodes a plant‐specific, highly conserved nuclear protein. The loss of *TAC4* function leads to a significant increase in the tiller angle. *TAC4* can regulate rice shoot gravitropism by increasing the indole acetic acid content and affecting the auxin distribution. A sequence analysis revealed that *TAC4* has undergone a bottleneck and become fixed in *indica* cultivars during domestication and improvement. Our findings facilitate an increased understanding of the regulatory mechanisms of tiller angle and also provide a potential gene resource for the improvement of rice plant architecture.

## Introduction

Plant architecture is defined as the morphological characteristics and three‐dimension spatial arrangement of plant organs (Reinhardt and Kuhlemeier, 2002). Tiller angle is an important component of plant architecture and has a great influence on the grain yield of rice (*Oryza sativa* L.). Too large or small a tiller angle exerts adverse effects on rice yield (Wang and Li, 2008), and a suitable tiller angle is very important for the growth and grain yield of rice. Therefore, tiller angle has long attracted the attention of breeders and plant biologists, and the elucidation of the mechanisms controlling tiller angle could aid in the development of high‐yielding rice varieties.

In recent decades, several genes affecting rice tiller angle have been identified. *LAZY1* (*LA1*) controls rice tiller angle by regulating gravitropism, and the *la1*‐mutant displays a tiller‐spreading phenotype because the gravitropism was reduced (Li *et al*., 2007). HEAT STRESS TRANSCRIPTION FACTOR 2D (HSFA2D) is an upstream positive regulator of the *LA1*‐mediated asymmetric auxin distribution pathway (Zhang *et al*., 2018). *Loose Plant Architecture 1* (*LPA1*) regulates both tiller angle and leaf angle by influencing gravity perception and signal transduction (Wu *et al*., 2012). *PLANT ARCHITECTURE AND YIELD 1* (*PAY1*) influences plant height, tiller number and angle, and panicle architecture by affecting auxin’s polar transport and distribution (Zhao *et al*., 2015). *Tiller Angle Control 1* (*TAC1*) is a key regulator responsible for the different tiller angles between *japonica* and *indica* rice. A mutation in the 3ʹ‐splicing site of its fourth intron decreases the level of *tac1*, resulting in the compact plant architecture of *japonica* (Yu *et al*., 2007). *TAC1* has conserved functions in regulating the tiller or branch angle in peach (*Prunus persica*) trees, *Arabidopsis thaliana*, wheat (*Triticum aestivum*) and *Miscanthus sinensis* (Dardick *et al*., 2013; Waite and Dardick, 2018; Zhao *et al*., 2014). *TAC3* and *D2* regulate tiller angle in rice cultivars together with *TAC1* (Dong *et al*., 2016). *PROSTRATE GROWTH 1* (*PROG1*), encoding a single Cys2–His2 zinc‐finger protein, plays an important role in rice domestication, and the artificial selection of *PROG1* results in great changes in rice plant architecture, resulting not only in the growth habit change from prostrate to erect growth, but also in improved rice yields (Jin *et al*., 2008; Tan *et al*., 2008). A 110‐kb deletion on the short arm of chromosome 7, which is closely linked to *PROG1*, promotes the critical transition from the prostrate growth and resulting low yield of wild rice to the erect growth and resulting high yield of cultivated rice (Wu *et al*., 2018). *TILLER INCLINED GROWTH 1* (*TIG1*), encoding a TCP transcriptional activator, controls tiller angle by regulating cell elongation during the domestication of *indica* rice (Zhang *et al*., 2019). Although such key genes controlling tiller angle have been characterized, the molecular mechanisms regulating the tiller angle of rice remain largely unknown.

In this study, we identified a novel gene *Tiller Angle Control 4* (*TAC4*) that participates in the regulation of tiller angle. *TAC4* encodes an evolutionarily conserved protein with unknown function(s). A mutation in *TAC4* decreased the endogenous auxin content, ultimately leading to reduced gravitropism and a tiller‐spreading phenotype.

## Results

### A *tac4* mutant displays a greater tiller angle phenotype

**Figure 1:**
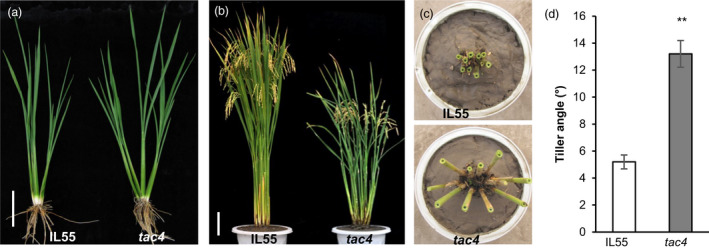
Phenotypic characterization of the *tac4* mutant. (a, b) Comparison of the phenotype between IL55 (left) and *tac4* (right) at the tillering (a) and ripening (b) stages. Scale bar, 10 cm (a). Scale bar, 20 cm (b). (c) Comparison of the tiller angle between IL55 (above) and *tac4* (below). (d) Tiller angle at the ripening stage. Error bars indicate SEM, *n* = 15. Student’s *t*‐tests were used to generate *P* values (** *P* < 0.01).

To identify novel genes that regulate rice tiller angle, the introgression line IL55 harbouring the *tac1* allele (Aida *et al*., 1997), which displays an extremely compact tiller angle in the *indica* rice variety IR24 background, was mutagenized with ethyl methane sulphonate to generate a mutant library for screening plants with large tiller angles. One such mutant, *tac4,* was identified. During both the vegetative and reproductive stages, *tac4* always exhibited a greater tiller angle than IL55 (Figure 1a and b, and Figure S1), and at the heading stage, the tiller angle was ~13.2° in the *tac4* mutant, while it was only ~5.2° in the IL55 (Figure 1c and d). The heights of *tac4* plants were reduced ~20% compared with those of IL55 and mainly resulted from the shortened uppermost internode (Figure S2a–c). Observations of longitudinal sections of the uppermost internode revealed that cell lengths were significantly decreased in *tac4*, indicating that the decreased cell elongation could be the main cause of the reduced *tac4* plant height (Figure S2d). We found that the panicle length was shorter, and the primary and secondary branches and the grain number per panicle were significantly reduced, compared with IL55 (Table S1). The grain length of *tac4 was* shorter than that of IL55, and the mean 1000‐grain weight was less (Figure S3). These observations suggest that *TAC4* is pleiotropic, regulating multiple important agronomic traits.

### Map‐based cloning of *TAC4*

To clone *TAC4*, the *tac4* mutant and a compact *japonica* rice variety Suweon392 were crossed to generate a mapping population. The F_1_ plants from the cross displayed smaller tiller angles, similar to the IL55 phenotype (Figure S4). Of 486 F_2_ plants, 115 showed the *tac4*‐like phenotype, and 371 showed the IL55‐like phenotype, fitting the 3:1 segregation ratio (*χ* ^2^ [3:1] = 0.045 < $\chi_{0.05,1}^{2}$ = 3.84). Thus, the *tac4*‐mutant phenotype appears to be controlled by a single recessive gene.

**Figure 2:**
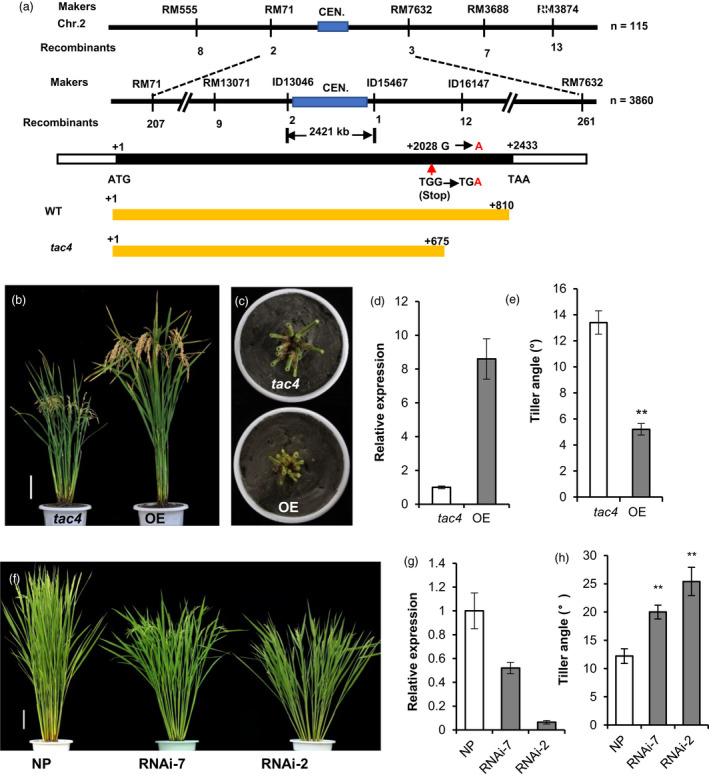
Cloning and functional confirmation of *TAC4*. (a) Fine mapping and a schematic representation of *TAC4*. Black boxes indicate the coding sequences, and white boxes indicate the untranslated regions. (b–e) The phenotypes of the *TAC4*‐overexpression transgenic plants. Plant architecture (b), tiller angle (c), the relative expression levels of *TAC4* (d) and data on the tiller angle (e). (f–h) The phenotypes of the *TAC4* RNAi transgenic plants. Tiller angle (f), the relative expression levels of *TAC4* (g) and data on the tiller angle (h). Error bars indicate SEM, *n* = 15. Student’s *t*‐tests were used to generate *P* values (** *P* < 0.01).

Using 115 *tac4*‐like plants from the F_2_ population, the *TAC4* locus was mapped to a region near the centromere of chromosome 2, between the simple sequence repeat markers RM71 and RM7632. To further delimit the *TAC4* locus, a large population containing about 15, 000 F_2_ plants was generated. Then, the *TAC4* locus was mapped within a 2421‐kb genomic region between the ID13046 and ID15467 markers, which is just right of the centromeric region (Figure 2a). Within this region, there are 156 predicted genes in the rice genome annotation database (http://rice.plantbiology.msu.edu). To identify the mutated gene, all the coding sequences of the 156 predicted genes were sequenced and compared between IL55 and *tac4*, and only one single nucleotide change, G to A, was found. It occurred at position + 2028 bp in the exon of LOC_Os02g25230 and resulted in a premature stop codon, which led to the encoded protein being truncated, with the C‐terminal 135 amino acids missing in the *tac4* mutant (Figure 2a).

The function of LOC_Os02g25230 was further confirmed by genetic transformation. All the transgenic plants overexpressing the full‐length coding sequence of LOC_Os02g25230 (derived from IL55) in the *tac4*‐mutant background displayed the small tiller angle phenotype, similar to IL55 (Figure 2b–e). Using RNA interference (RNAi), we generated LOC_Os02g25230 knockdown plants in the *japonica* cultivar Nipponbare background. The RNAi transgenic plants exhibited greater tiller angles compared with control plants, indicating that the decreased expression of LOC_Os02g25230 significantly increased tiller angle (Figure 2f–h). Therefore, we concluded that LOC_Os02g25230 corresponds to the *TAC4* gene and that the expression level of *TAC4* was negatively correlated with tiller angle.

### Expression pattern and subcellular localization of TAC4

**Figure 3:**
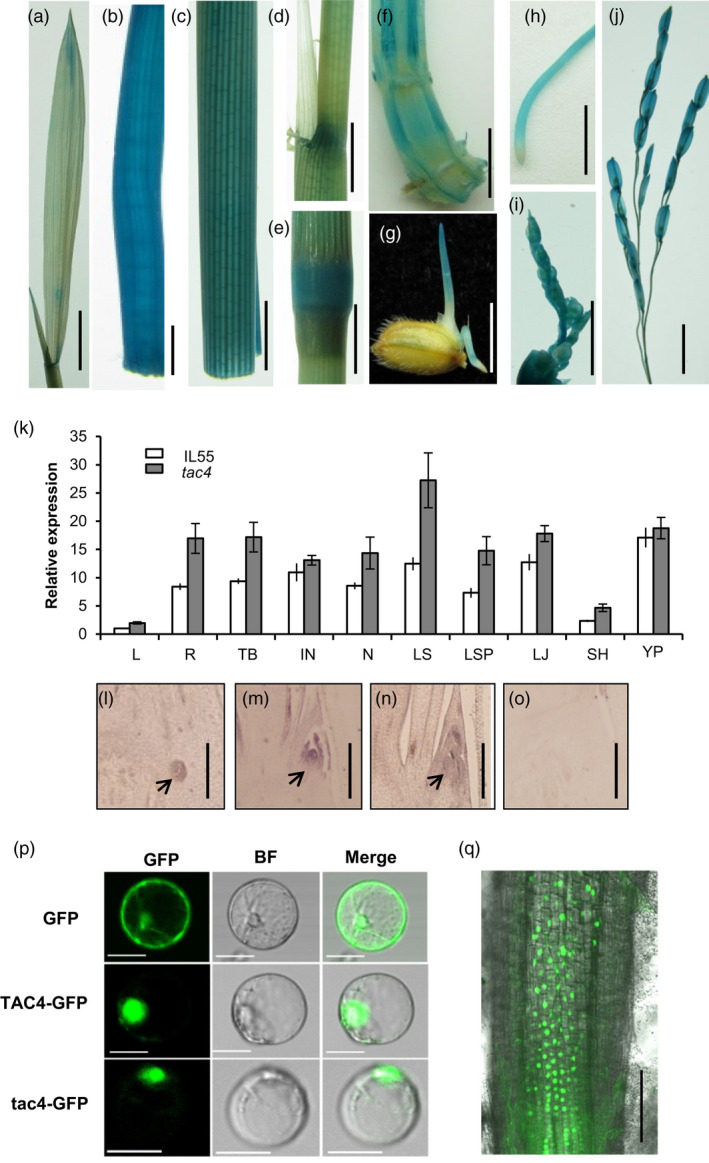
*TAC4* expression pattern and its product’s subcellular localization. (a–j) GUS activity levels in various organs. Leaf, (a); internode, (b); leaf sheath (c); leaf sheath pulvinus, (d); node, (e); tiller base, (f); coleoptile, (g); root, (h); young panicle (2 cm), (i); and young panicle (5–10 cm), (j). Scale bars, 1 cm. (k) The relative expression levels of *TAC4* in various organs. L, leaf; R, root; TB, tiller base; IN, internode; N, node; LS, leaf sheath; LSP, leaf sheath pulvinus; LJ, lamina joint; SH, spikelet hull and YP, young panicle. (l–o) RNA in situ hybridization. Expression patterns of *TAC4* were measured in the tiller bases at 30‐day after sowing. Black arrowheads indicate the positions of the tiller primordium (l) and axillary bud (m and n) in the tiller base. The sense probe was hybridized and used as the negative control (o). Scale bars, 200 μm. (p–q) Subcellular localizations of TAC4 and tac4. The TAC4‐GFP and tac4‐GFP fusion proteins are present within the nuclei in rice protoplasts (p) and in the roots of *p35S:TAC4–GFP* transgenic plant (q). Scale bar, 20 μm (p); Scale bar, 100 μm (q).

To determine the temporal and spatial expression patterns of *TAC4*, we introduced a construct, consisting of a 2175‐bp *TAC4* promoter fused to the *GUS* reporter gene, into the *japonica* cultivar Nipponbare. GUS staining of the transgenic plants indicated that *TAC4* was expressed in almost all the organs, including root, culm, leaf sheath, tiller base and young panicle (Figure 3a–j). Consistent with the GUS staining data, a quantitative real‐time PCR analysis also showed that *TAC4* was ubiquitously expressed in various rice organs, with the most abundant expression occurring in the young panicle, lamina joint, leaf sheath pulvinus and internode (Figure 3k). *TAC4* mRNA in situ hybridization showed that a high *TAC4* transcript abundance could be detected in the axillary and shoot apical meristem at the tillering stage (Figure 3l–o). RNA‐sequencing data showed *TAC4* transcripts in both the lower and upper sides of tiller bases, and the expression levels were similar (Figure S5). The high expression of *TAC4* was also detected in the tiller base during the whole growth period (Figure S6). This *TAC4* expression pattern is consistent with its role in the control of tiller angle.

The *TAC4* gene was predicted to encode a novel protein. To elucidate the cellular localization of the TAC4 protein, we fused green fluorescence protein (GFP) to the C termini of TAC4 and tac4. The fluorescent signal of the TAC4‐GFP fusion was detected in the nuclei of rice leaf protoplasts (Figure 3p) and in the roots of transgenic plant (Figure 3q), suggesting that the TAC4 protein may function in the nucleus. The fluorescent signal of tac4‐GFP was also detected in the nucleus (Figure 3p). Thus, the loss of 135 residues from the C terminus of TAC4 resulted in the protein’s loss of function, yet it did not change the cellular localization.

### *TAC4* regulates tiller angle by influencing shoot gravitropism

**Figure 4:**
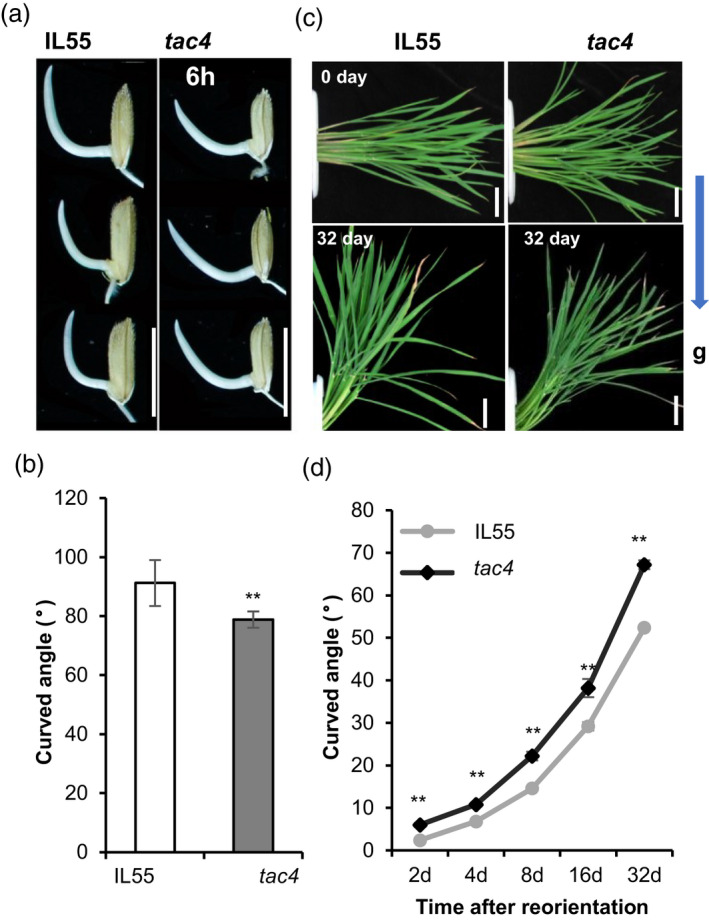
Gravitropic analysis of IL55 and *tac4*. (a and b) Coleoptiles of IL55 and *tac4* plants after 6 h of gravistimulation. Scale bars, 1 cm. (c and d) Stem curvature of IL55 and *tac4* plants at the tillering stage after 32‐ day of gravistimulation. Scale bars, 10 cm (c). Kinetic analysis of stem curvature upon gravistimulation (d). Error bars indicate SEM, *n* = 10. Student’s *t*‐tests were used to generate *P* values (** *P* < 0.01).

Plant gravitropic responses have great influences on the tiller angle. To determine whether *TAC4* regulates tiller angle by regulating shoot gravitropism, we first examined the gravity responses of coleoptiles using 1‐cm‐long young shoots of the IL55 and *tac4* plants grown under continuous dark conditions. The coleoptiles’ gravitational response in the *tac4* mutant was significantly reduced compared within the IL55 (Figure 4a and b). Next, we used 70‐day‐old paddy‐field grown plants at their tilling stage to conduct the gravitational response assay. The results were similar to the coleoptile gravitational response, and the shoots of the *tac4* mutant were less sensitive to the gravitational response. The difference in tiller curved angle between *tac4* and IL55 appeared after a 2‐day gravitational treatment, and the difference in tiller curved angle increased in tandem with the number of days of gravitational treatment. The tiller curved angle was about 70° in IL55 and 50° in *tac4* after a 32‐day gravitational treatment (Figure 4c and d). Thus, *TAC4* regulated the rice tiller angle by influencing shoot gravitropism.

### *TAC4* regulates shoot gravitropism by affecting the auxin content and distribution

**Figure 5:**
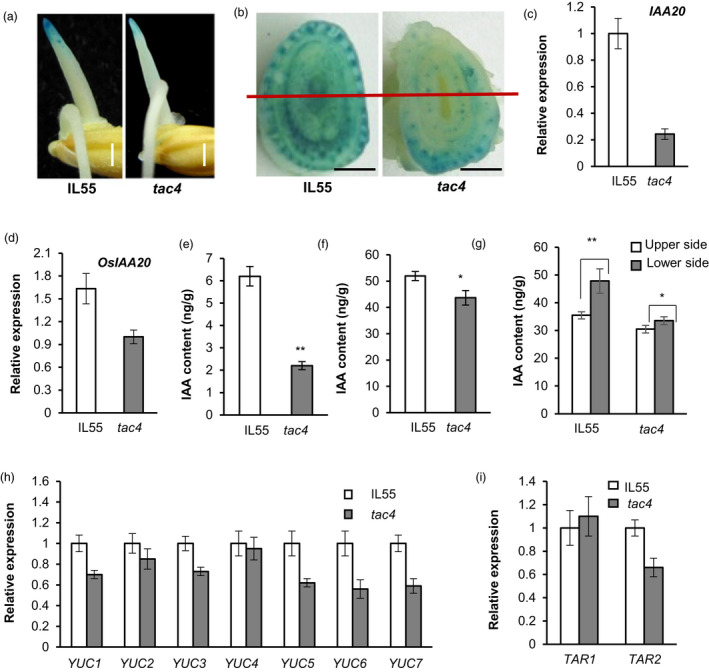
*TAC4* regulates the auxin level. (a) *DR5:GUS* expression patterns in dark‐grown coleoptiles. Scale bars, 1 mm. (b) *DR5:GUS* expression patterns in the tiller bases. Scale bars, 2.5 mm. (c and d) The expression levels of *OsIAA20* in the tips of dark‐grown coleoptiles (c) and in the tiller bases (d). (e and f) The IAA contents in the tips of dark‐grown coleoptiles (e) and in the tiller bases (f). (g) IAA contents in the lower and upper sides of the tiller base. (h–i) Expression levels of *YUC*s (h) and *TAA* (i) family genes. Student’s *t‐*tests were used to generate *P* values, * *P* < 0.05, **P < 0.01.

The asymmetrical distribution of auxin plays predominant roles in gravitropism and tiller angle. To characterize the involvement of auxin in *TAC4*‐mediated gravitropism, we investigated the endogenous auxin distribution by analysing GUS expression levels in the transgenic plants using the auxin reporter *DR5:GUS*. The GUS signals at the apical coleoptile and tiller base of the *tac4* mutant were markedly less intense than those of IL55 (Figure 5a and b), indicating that the endogenous auxin in the apical coleoptile and tiller base of *tac4* plants decreased compared with in the IL55 plants. We also examined the expression levels of the endogenous auxin‐response marker gene *IAA20* in the coleoptile and tiller base in the IL55 and *tac4* plants. Compared with IL55, the transcript abundance of *OsIAA20* in the *tac4* mutant was obviously reduced (Figure 5c and d). These results indicated that the endogenous auxin content was decreased in *tac4*. Consequently, we further determined the contents of endogenous auxins and found that the endogenous indole‐3‐acetic acid (IAA) contents in the coleoptile and tiller base of *tac4* were significantly lower than those in IL55, which was consistent with the expression levels of *DR5:GUS* and *IAA20* (Figure 5e and f). Furthermore, the difference in the IAA content between the lower and upper sides in *tac4* was less than that in IL55 (Figure 5b and g). Thus, *tac4* may regulate tiller angle by decreasing the endogenous auxin content and changing its asymmetric distribution.

To determine whether the weak gravitropic responses of *tac4* were caused by auxin defects, we examined the shoot gravitropic responses of seedlings upon treatment with auxin. We found that the application of an auxin IAA or NAA (1‐Naphthaleneacetic acid potassium salt) enhanced the gravitropic responses of the *tac4* seedlings compared with non‐treated seedlings (Figure S7). Although auxin applications improved the gravitropic responses of the *tac4* seedlings, they showed weaker gravitropic responses than IL55 under control conditions (Figure S7). Thus, results auxin defects may be a main reason for the weak gravitropic responses of *tac4*, and the application of auxin could partially rescue this phenotype.

IAA is mainly synthesized from the amino acid tryptophan in two consecutive chemical steps. The auxin biosynthetic genes *YUCCA* (*YUC*) and *TRYPTOPHAN AMINOTRANSFERASE OF ARABIDOPSIS* (*TAA)* play key roles in the tryptophan‐dependent auxin synthetic pathway (Stepanova *et al*., 2008; Zhao *et al*., 2001). Therefore, we investigated the transcript abundance levels of the *YUC* (*YUC1*–*7*) and *TAA* (*TAR1* and *TAR2*) family genes in IL55 and *tac4*. The expression levels of most IAA synthetic genes were lower in the coleoptiles of the *tac4* mutant than those in IL55, especially *YUC5–7* (Figure 5h and i). Therefore, *TAC4* could affect the endogenous IAA content by influencing the expression levels of IAA synthetic genes in rice.

### *TAC4* encodes an evolutionarily conserved plant‐specific protein

A sequence analysis revealed that *TAC4* harbours only one exon in its coding region. To explore the evolutionary history of *TAC4*, we analysed the genetic diversity of the *TAC4* coding region by comparing *TAC4* alleles from a representative panel of 40 accessions of wild rice (*Oryza rufipogon*), 199 cultivated varieties, including 112 *indica* varieties (*O*.* sativa* ssp. *indica*) and 87 *japonica* varieties (*O*.* sativa* ssp. *japonica*), collected from 19 rice‐growing regions across the world (Table S2). We found 26 single nucleotide polymorphisms (SNPs) in the *TAC4* locus of wild accessions, but only three SNPs in cultivated rice. Among these SNPs, only eight SNPs in the wild accessions and one SNP (1793T > C) in cultivated rice resulted in amino acid substitutions. Based on the amino acid changes in these accessions, seven haplotypes (Hap1–7) were identified in wild rice accessions, and only two haplotypes (Hap1 and Hap2) were found in cultivars (Figure S8a). Among the 199 cultivars, the vast majority of *indica* rice cultivars (91.96%) and 35.63% of *japonica* rice cultivars were Hap1 at the *TAC4* locus, with the remaining 64.37% of *japonica* rice cultivars being Hap2 (Figure S8b). An analysis of the nucleotide diversity in *TAC4* demonstrated that the sequence diversity was only 0.00052 in *O*.* sativa*, less than half of that in *O*.* rufipogon* (*π* = 0.00114), with an even more significantly decreased nucleotide diversity (*π* = 0.00015) in the *indica* cultivars. Tajima’s *D* values suggested that there was no strong directional selection at the *TAC4* locus in cultivated rice (Table S3). The fixation index (*F*_st_) analysis showed a greater genetic differentiation between *indica* and *japonica* (*F_st_* = 0.50) than between *O*.* rufipogon* and *japonica* (*F*_*st*_ = 0.29) or between *O*.* rufipogon* and *indica* (*F_st_* = 0.24; Table S4). To exclude the potential effects of a selective sweep on diversity reduction at *TAC4*, we examined the nucleotide diversity of 14 loci in a 1.5‐M region surrounding the gene in 1,617 cultivated varieties (including 698 *japonica* rice and 919 *indica* rice) and 53 wild rice accessions based on sequencing data reported previously (Xu *et al*., 2012). We found that the ratios of nucleotide diversity around the *TAC4* locus between the two subspecies of *Oryza sativa* and wild rice were not significantly different (Figure S9), indicating that there was no distinct selective sweep surrounding the genomic region of *TAC4*. Thus, the fixation of Hap1 and Hap2 in cultivars, especially Hap1 in *indica*, is most likely due to the result of a bottleneck founder effect during rice domestication and improvement.

A BlastP analysis revealed that *TAC4* encodes a novel protein without any functionally known domains (http://smart.embl.de). The sequence analysis showed that LOC_Os04g19140 and TAC4 have high similarity levels in rice. The analysis also showed that homologs of *TAC4* are widely found in plants, but not in animals and yeasts (Figure S10). TAC4 homologs from *Sorghum bicolor*, *Zea mays* and *Setaria italic* share high levels of sequence similarity with TAC4 (84.2% or higher amino acid‐level sequence identities; Figure S11), and even in moss, the non‐flowering plant, the amino acid‐based sequence identity level reached 39.8% (Figure S10). Thus, *TAC4* appears to be an ancient evolutionarily conserved plant‐specific gene.

### *TAC4* and*TAC1* act in distinct pathways to regulate tiller angle

*TAC1* is a major quantitative trait locus responsible for the tiller angles, and its expression level is positively correlated with the tiller angle (Yu *et al*., 2007). To determine whether *TAC4* regulates tiller angle in the same pathway as *TAC1,* we generated a set of near‐isogenic lines and pyramiding lines. We found that the order of tiller angle was as follows: *TAC1/tac4* (27.8°) > *TAC1/TAC4* (18.2°) > *tac1/tac4* (14.6°) > *tac1/TAC4* (4.4°), and *TAC1/tac4* exhibited the greatest tiller angle (Figure S12). Thus, *TAC4* and *TAC1* participate in distinct pathways to regulate tiller angle.

## Discussion

As a key component of plant architecture, tiller angle affects plant density, photosynthetic efficiency, lodging and disease resistance, as well as playing an important role in determining rice grain yield. Although several genes regulating tiller angle have been identified, the regulatory mechanism of rice tiller angle is still unknown to a large extent. In this study, we identified the novel gene *TAC4*.*TAC4* encodes a plant‐specific protein without any known conserved domains that controls tiller angles in rice. The substitution of an amino acid in *TAC4* reduced the gravitational response by decreasing the auxin level and changing its distribution, which ultimately led to a greater tiller angle and reduced plant height. A sequence analysis suggested that *TAC4* may be an evolutionarily conserved gene that plays an important role in regulating plant architecture.

### *TAC4* regulates plant architecture by affecting the auxin content and distribution

The asymmetrical distribution of auxin plays a key role in the gravitropic responses of plants, which play predominant roles in regulating tiller angle. Additionally, the asymmetric distribution is determined by the polar transport and synthesis of auxin; and therefore, the factors controlling the polar transport and synthesis of auxin generally affect the tiller angle. For example, changing the expression levels of auxin efflux transporters alters polar auxin transport, which alters the rice tiller angle (Chen *et al*., 2012; Xu *et al*., 2005). The loss of *LA1* function increases the polar transport of auxin, alters the gravitropism of the plant, and ultimately leads to extremely spread tillers (Li *et al*., 2007). The transcripts of some auxin synthesis‐related genes are enriched in the responses of rice shoots to gravistimulation (Zhang *et al*., 2018). In this study, the mutation of *TAC4* decreased the expression levels of auxin synthetic *YUC*s and *TAR2*, resulting in a decrease in the auxin content, and changed the auxin distribution pattern (Figure 5), which in turn affected the shoot gravitropic responses of plants (Figure 4). This ultimately affected tiller angle. Therefore, we concluded that *TAC4* regulates plant architecture by affecting the auxin content and distribution.

### *TAC4* has pleiotropic effects on plant architecture and yield‐related traits

Some genes that control important agronomic crop traits have pleiotropic effects. For example, *RGA1/D1* determines plant height, panicle architecture, leaf shape and grain size in rice (Fujisawa *et al*., 1999; Ueguchi‐Tanaka *et al*., 2000). *Ghd7* regulates plant height, grain number and heading date (Xue *et al*., 2008). *GAD1* regulates grain number, grain size and awn development (Jin *et al*., 2016). *FZP* controls panicle secondary branch number, grain number, plant height, grain length and flag leaf width (Bai *et al*., 2017; Huang *et al*., 2018). In this study, the mutant *tac4* plants also exhibited reduced plant heights, shorter grain lengths, decreased grain weights, but had greater tiller angles, compared with IL55 (Figure 1 and Figure S3), indicating that *TAC4* is also a pleiotropic gene, which modulated tiller angle and also influenced plant height and grain size.

Auxin is an important plant hormone and plays a key role in every aspect of plant growth and development. Changes in the auxin content and distribution pattern can lead to effects on multiple characteristics, such as plant height, tiller angle, leaf angle, root length and grain size (Li *et al*., 2007; Liu *et al*., 2015; Wu *et al*., 2012; Zhao *et al*., 2015). The endogenous auxin content was decreased, and its distribution pattern was changed in *tac4* (Figure 5). Therefore, we speculated that *TAC4* regulates rice plant architecture and yield‐related traits by affecting the endogenous auxin content and its asymmetrical distribution.

### Using mutants may be an effective way to identify genes in the centromere‐adjacent regions

Map‐based cloning and genome‐wide association studies are powerful methods for identifying novel genes in the chromosome, and a large number of genes that control different traits have been identified in plants. The major principles behind map‐based cloning and genome‐wide association studies are the linkage and recombination of chromosomes. The recombination rates of regions adjacent to the centromere are very low. For example, the recombination density of the ~2.5 Mb centromeric core region is ~50 times lower than that of the chromosome arm (Yelina *et al*., 2012), which is the reason why genes near the centromere are difficult to locate. However, some genes are located in the pericentromeric regions in eukaryotes (Liao *et al*., 2018), and essential genes are preferentially located in regions near the centromere (Taxis *et al*., 2005). In rice, only a few genes located in the centromere‐adjacent regions have been cloned. For example, *LA1*, the first identified tiller angle regulator, similar to *TAC4*, is located near the centromeric region (Li *et al*., 2007). *TAC4* and *LA1* were both identified by analysing mutants. With the rapid development of DNA sequencing technologies, it is now easy to identify and locate genes controlling the mutant traits using MutMap and other methods (Abe *et al*., 2012). Therefore, using mutants may be an effective way to identify important genes near the centromere.

### *TAC4* and *TAC1* were fixed in *indica* and*japonica* subspecies, respectively, during the differentiation of cultivated rice

Domestication is a complex, cumulative evolutionary process in which humans modify a wild plant species to meet their needs and promote the development of society (Doebley *et al*., 2006). During domestication, the genetic diversity levels of the cultivars decrease to varying degrees compared with those of the wild ancestors (Tanksley and McCouch, 1997). The population bottleneck and intense selections for agronomic traits are two main reasons for the induction of genetic diversity (Eyre‐Walker *et al*., 1998; Zeder *et al*., 2006; Zhu *et al*., 2007). The identified domesticated genes of rice have undergone strong artificial selection (Gu *et al*., 2015; Hua *et al*., 2015; Ishii *et al*., 2013; Jin *et al*., 2008; Konishi *et al*., 2006; Li, *et al*., 2006; Lin *et al*., 2007; Luo *et al*., 2013; Tan *et al*., 2008; Zhu *et al*., 2011; Zhu *et al*., 2013;), but few identified genes have undergone the bottleneck founder effect during rice domestication. Chen et al. found that *FUWA*, which mainly regulates panicle architecture, and grain shape and weight, has been subjected to the bottleneck effect (Chen *et al*., 2015). In this study, we found that the nucleotide diversity level of *TAC4* in cultivated rice, especially in *indica* rice, was significantly lower than the levels of its ancestors. Haplotype and fixation index analyses revealed that *TAC4* might have also been subjected to a bottleneck during rice domestication and improvement (Figure S8) and become fixed in *indica* rice. *TAC1*, which is an important quantitative trait locus controlling tiller angle, is fixed in *japonica* rice (Jiang *et al*., 2012; Yu *et al*., 2007), indicating that *TAC4* and *TAC1* were fixed in *indica* and *japonica* subspecies, respectively, during the differentiation of cultivated rice.

Tiller angle determines plant density and largely affects the grain yield per area. The identification of novel genes regulating tiller angle is of significance for further improving rice plant architecture and grain yield. The identification of *TAC4* not only helps increase our understanding of the regulatory mechanisms involved in establishing tiller angle, but it also provides a favourable gene for improving the plant architecture and grain yield of rice.

## Materials and methods

### Plant materials and growth conditions

IR24, IL55, the *tac4* mutant, Suweon392, the segregation population for mapping and transgenic rice plants were grown in paddy fields in Beijing in the summer or in Hainan Province in the winter. A total of 239 accessions, 40 accessions of *O*.* rufipogon*, 112 of *indica* varieties and 87 of *japonica* varieties, used in this study were grown in paddy fields in Beijing in the summer. Seedlings were grown in soil or on 1% agar plates at 28°C under a 16‐h light/8‐h dark cycle.

To determine whether *TAC4* regulates tiller angle in the same pathway as *TAC1,* we generated a set of near‐isogenic lines and pyramiding lines. IL55, the wild type used in this study, is an introgression line in the background of IR24 (Aida *et al*., 1997) and harbours the *tac1/TAC4* genotype. The mutant *tac4* harbours the *tac1/tac4* genotype. IR24 harbours the *TAC1/TAC4* genotype. Plants harbouring the *TAC1/tac4* genotype were selected from a cross between the *tac4* mutant and IR24.

The *TAC4* alleles in Nipponbare, Suweon392 and IL55 are *TAC4^Hap2^*, *TAC4^Hap2^* and *TAC4^Hap1^*, respectively, and all three lines carry *tac1* allele.

### Primers

The primers used in this study are listed in Table S5.

### Map‐based cloning of *TAC4*

To map the *TAC4* locus, the *tac4* mutant was crossed with Suweon392 (compact *japonica* rice variety), and the resulting F_1_ plants were self‐crossed to generate an F_2_ mapping population. Then, 115 *tac4*‐like plants from the 436 F_2_ progeny were selected for rough mapping, and the *TAC4* locus was mapped to a region near the centromere of chromosome 2, between the simple sequence repeat markers RM71 and RM7632. To further delimit the *TAC4* locus, a large mapping population, containing ~15 000 F_2_ plants, was generated and 3600 recessive individuals were used for fine mapping.

### Genetic confirmation

The entire coding sequence of the *TAC4* cDNA (derived from IL55) was inserted into the vector pCAMBIA1301 driven by the maize Ubiquitin promoter to form the *TAC4* overexpression construct. The construct *TAC4‐*RNAi was generated by the insertion of a hairpin sequence containing two 300‐bp cDNA inverted‐repeat fragments targeting the sequence of *TAC4* into *p* *TCK303*, driven by the maize Ubiquitin promoter. All the resulting plasmids were transformed into *Agrobacterium tumefaciens* strain EHA105 and were then introduced into *tac4*‐mutant calli or Nipponbare calli.

### Subcellular localization of TAC4

The constructs *p35S:TAC4–GFP* and *p35S:tac4–GFP*, which contained TAC4 and tac4, respectively, fused with GFP and driven by a 35S promoter, were constructed and introduced into Nipponbare calli or rice seedling protoplasts. Then, GFP signals were visualized using a Carl Zeiss LSM510 Meta confocal laser scanning microscope.

### Quantitative RT‐PCR analysis

For the quantitative RT–PCR analysis, total RNA was extracted using an RNeasy plant mini kit (Qiagen, http:// www.qiagen.com/). First‐strand cDNA was synthesized using ~2 μg of total RNA in a 20 μL volume with an Oligo (dT)18 primer and M‐MLV reverse transcriptase (Promega, San Luis Obispo, CA). PCR was performed in triplicate for each sample with three independent biological replicates. The rice *Actin* gene was used as an internal control. The quantitative RT–PCR was performed on a CFX96 Real‐Time System (Bio‐Rad, Hercules, CA). Diluted cDNA was amplified using SYBR Green Master Mix (Applied Biosystems, Foster City, CA). Each set of experiments was repeated at least three replications. The rice *Actin* gene was used as the internal control, and the gene expression levels in three biological replicates were calculated using the ΔΔCt (threshold cycle) method.

### RNA *in situ* hybridization

The 2‐kb promoter upstream of the *TAC4* translation start site was inserted into the vector *p* *CAMBIA1381* GUS to form the *pTAC4:GUS* construct. Then, the *pTAC4*:*GUS* construct was transformed into the *japonica* variety Nipponbare, and the resulting transgenic plants were analysed using a standard GUS staining assay (Scarpella *et al*., 2003).

RNA *in situ* hybridization was performed as described previously. After 30‐day of sowing, the tiller bases of IL55 were fixed in 3.7% (v/v) FAA solution, dehydrated, embedded in paraffin (Thermo Fisher Scientific, Scotts Valley, CA) and sliced into 8‐μm sections using a microtome (Leica RM2145, Burlingame, CA). Digoxigenin‐labelled RNA probes were prepared using a DIG RNA labelling kit (Roche, Basel, Switzerland). RNA hybridization and immunological detection of the hybridized probes were performed following the methods of Zhang *et al*. (2007).

### Measurement of free IAA content

IAA extraction and measurement were performed using liquid chromatography–mass spectrometry as described previously (Kowalczyk and Sandberg, 2001) with some modifications. The tips (0.2 cm in length) of 3‐day‐old dark‐grown coleoptiles or the tiller bases of 70‐day‐old plants were harvested and used for the assay. After extraction and purification, the samples were subjected to liquid chromatography–mass spectrometry analysis using a liquid scintillation counter (1450 MicroBeta TriLux, http://www.perkinelmer.com).

### Measurement of the tiller angle

The tiller angle was measured between the main culm and the outside tillers in 20 plants at 40 days after sowing, and every 7 days thereafter until 140 days after sowing. The tiller angles of the transgenic plants overexpressing *TAC4* and *TAC4*‐RNAi plants were measured at the heading stage.

### Analysis of plant gravitational responses

The gravitropic assay was carried out as described previously (Li *et al*., 2007) with some modification. The gravitational responses of coleoptiles were tested by measuring the coleoptile curved angle using 2‐day‐old seedlings grown at 28°C in the dark and rotated 90° every 2 h. The gravitational responses of shoots during the vegetative stage were tested by measuring the shoot curved angle of 70‐day‐old plants at different time points after reorienting by 90°. To determine whether auxin can rescue the weak gravitropic responses of *tac4*, 7‐day‐old seedlings grown in soil were treated with 5 mm IAA or 0.1 mm NAA and reoriented by 90° for up to 48 h.

### Sequence analysis

Nucleotide sequences of the coding regions were obtained from 40 accessions of wild rice (*O*.* rufipogon*) and 199 cultivars (*O*.* sativa*) from diverse geographical locations across Asia (Table S2). The number of polymorphic sites, number of haplotypes, mean proportion of pairwise differences per base pair (π) and the fixation index (*F*_st_) were calculated using DNASP version 5 (Rozas *et al*., 2003).

## Conflict of interest

The authors declare that they have no conflicts of interest.

## Author contributions

C.S. conceived and supervised this project; H.L. and H.S. performed most of the experiments; J. J. helped identify the mutant; X.S. provided material management support; L.T. supervised the experiments and gave technical assistance; H.S., C.S. and L.T. analysed the data and wrote the manuscript.

## Supporting information


**Figure S1** Kinetic analysis of tiller angle between IL55 and the *tac4* mutant.
**Figure S2** *TAC4* regulates plant height.
**Figure S3** *TAC4* regulates grain size and weight.
**Figure S4** Phenotypic characterization of F_1_ plants derived from a cross of IL55 and the *tac4* mutant
**Figure S5** The FPKM values of *TAC4* in RNA‐seq data of tiller bases of 70‐day‐old IL55 plants.
**Figure S6** Kinetic analysis of the transcript abundance of *TAC4*
**Figure S7** The gravity response of *tac4* can be rescued by an auxin application.
**Figure S8** *TAC4* variants in wild and cultivated rice.
**Figure S9** Comparison of diversity ratios surrounding the *TAC4 locus*.
**Figure S10** Phylogenetic tree of 25 homologs of TAC4 in land plants.
**Figure S11** Multiple alignment of TAC4 and its homologs.
**Figure S12** *TAC4*’s regulation of tiller angle is independent of *TAC1*.

**Table S1** Comparison of agronomic traits of IL55 and *tac4* plants.
**Table S2** Summary of the *TAC4* allele type in each wild strain and cultivar in the rice germplasm core collection.
**Table S3** Nucleotide diversity and Tajima’s *D* test.
**Table S4** The *F_st_* analysis at the *TAC4* locus.
**Table S5** Primers used in the study.
