# Supplementary material for: TAC4 controls tiller angle by regulating the endogenous auxin content and distribution in rice
Source: Plant Biotechnol J. 2020 Jul 20;19(1):64–73. doi: 10.1111/pbi.13440 (PMC7769243; doi:10.1111/pbi.13440)
Supplement: Supplementary file 1 — Figure S1 Kinetic analysis of tiller angle between IL55 and the tac4 mutant. Figure S2 TAC4 regulates plant height. Figure S3 TAC4 regulates grain size and weight. Figure S4 Phenotypic characterization of F1 plants derived from a cross of IL55 and the tac4 mutant Figure S5 The FPKM values of TAC4 in RNA‐seq data of tiller bases of 70‐day‐old IL55 plants. Figure S6 Kinetic analysis of the transcript abundance of TAC4 Figure S7 The gravity response of tac4 can be rescued by an auxin application. Figure S8 TAC4 variants in wild and cultivated rice. Figure S9 Comparison of diversity ratios surrounding the TAC4 locus. Figure S10 Phylogenetic tree of 25 homologs of TAC4 in land plants. Figure S11 Multiple alignment of TAC4 and its homologs. Figure S12 TAC4’s regulation of tiller angle is independent of TAC1. [file PBI-19-64-s002.pdf]

Figure S1

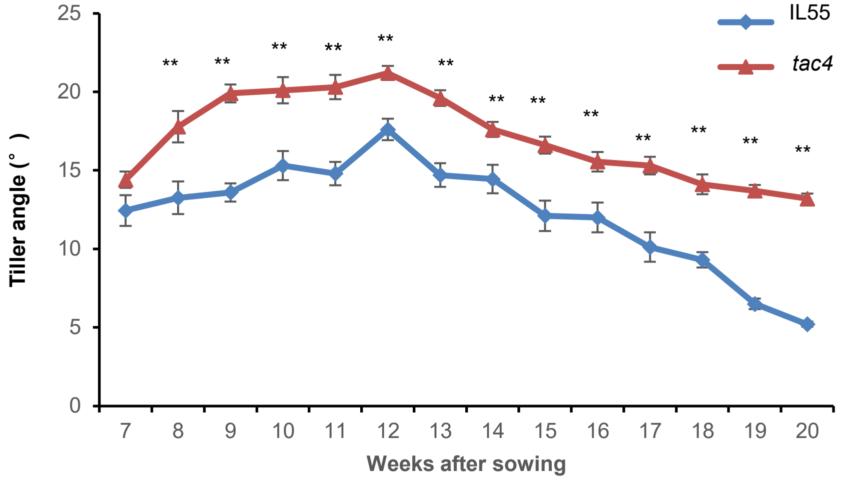

Figure S1. Kinetic analysis of tiller angle between IL55 and the *tac4* mutant. Error bars indicate SEM, *n* = 10. The double asterisks represent a significant difference determined by Student's *t*-test at $P < 0.01$.

Figure S2

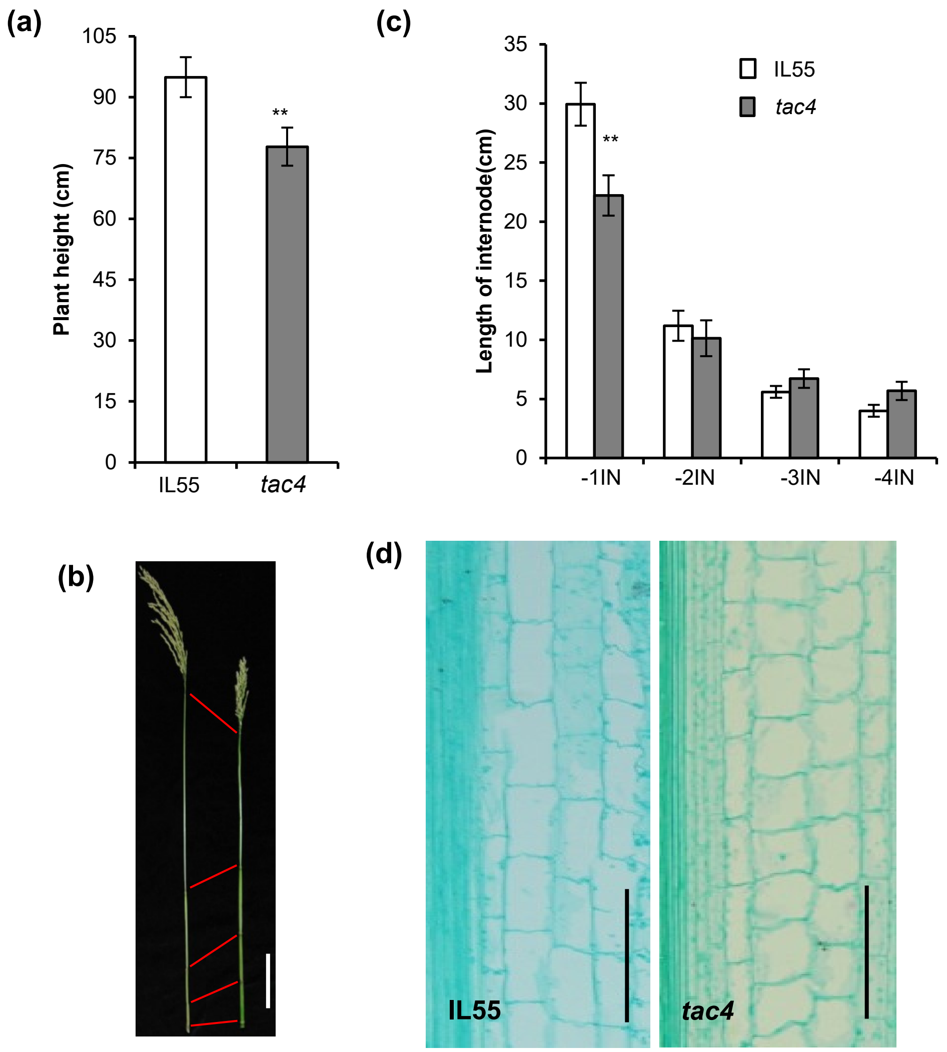

Figure S2. *TAC4* regulates plant height.

(a) Comparison of the plant height between IL55 and *tac4* mutant. (b-c) Internode length comparison. Scale bar, 10 cm. (d) Longitudinal sections of the uppermost internodes. Scale bar, 100 μm. Error bars indicate SD, $n = 20$. The double asterisks represent a significant difference determined by Student's t test at $P < 0.01$.

Figure S3

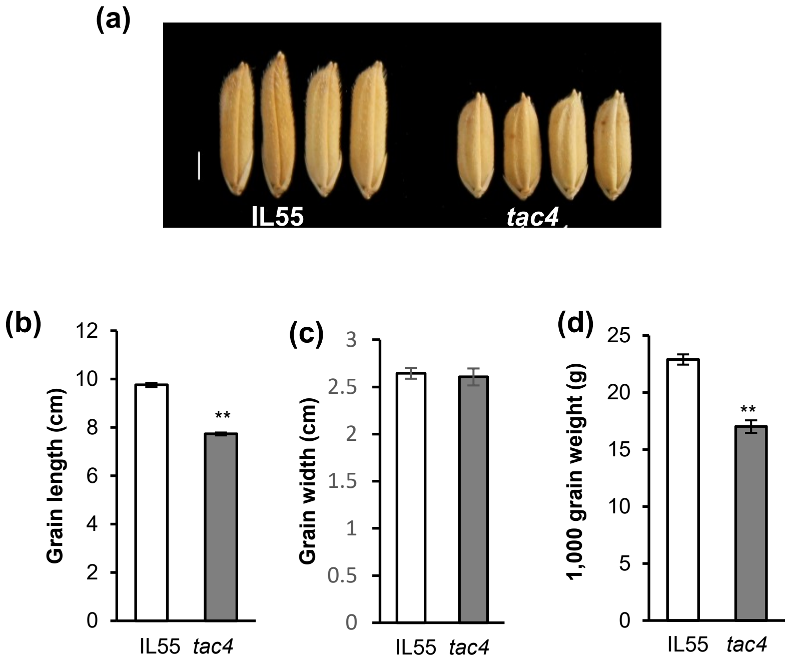

Figure S3. ***TAC4* regulates grain size and weight.**

(a) Grains of IL55 and *tac4*. Scale bar, 2 mm.

(b-d) Comparison of grain size and weight. The double asterisks represent a significant difference determined by Student's *t* test at $P < 0.01$.

Figure S4

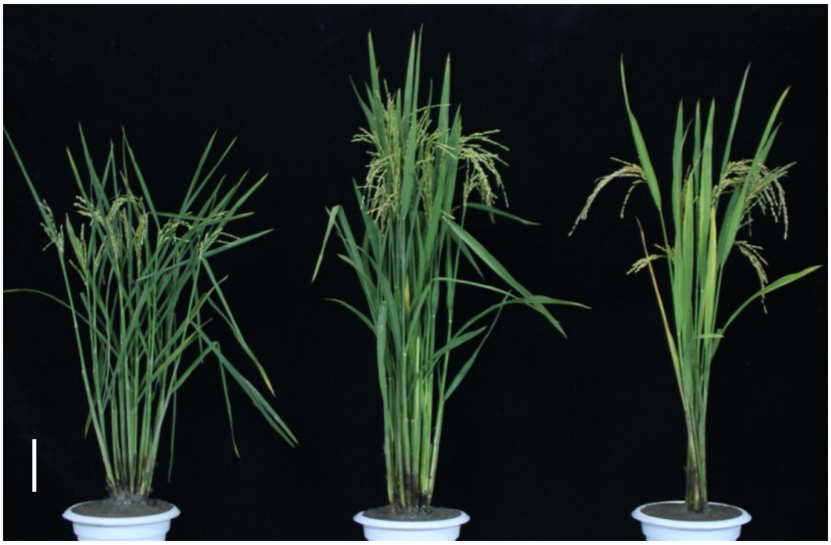

tac4	F_1	Suweon392

Figure S4. Phenotypic characterization of F_1 plants derived from a cross of between Suweon392 and the *tac4* mutant. Scale bar, 15 cm.

Figure S5

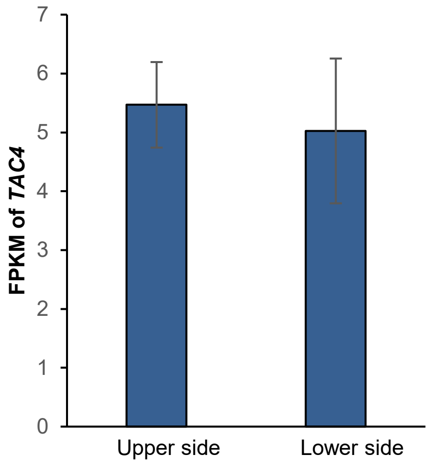

Figure S5. The FPKM values of *TAC4* in RNA-seq data of tiller bases of 70-day-old IL55. n=3

Figure S6

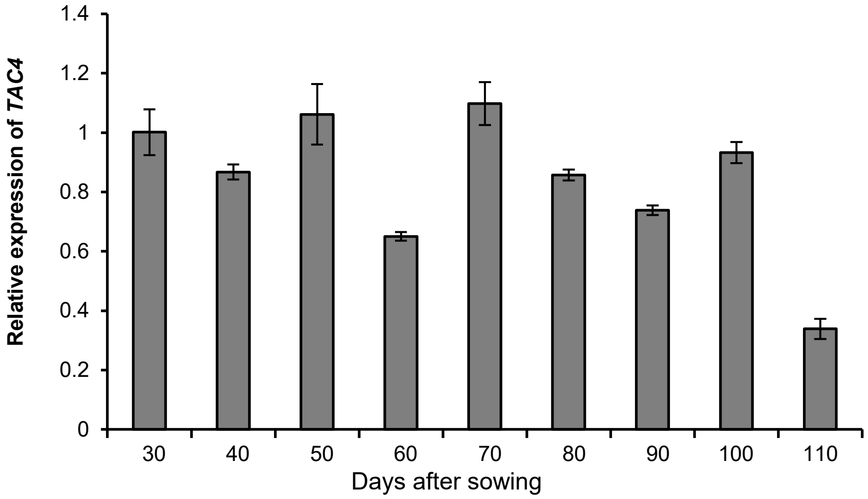

Figure S6. Kinetic analysis of the transcript abundance of *TAC4*. values are ± SD (*n* = 3).

Figure S7

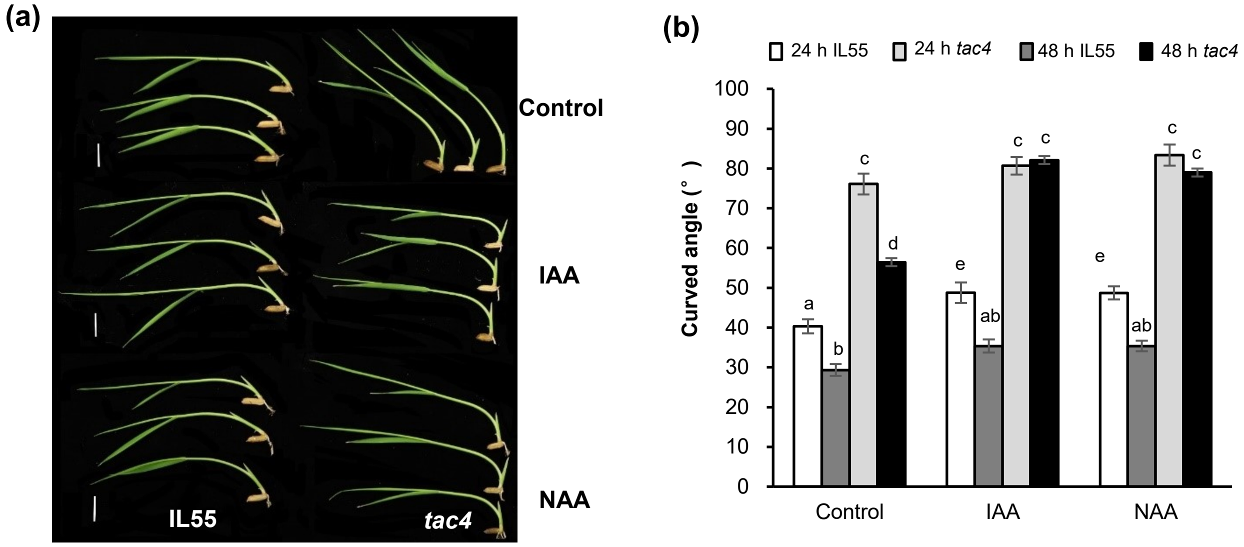

Figure S7. The gravity response of *tac4* can be rescued by an auxin application.

(a) Shoot curvature of IL55 and *tac4* at the seedling stage after 48 h of gravistimulation with or without application of auxin (IAA or NAA). Bar = 1 cm

(b) Shoot curvature comparisons after 24 h and 48 h of gravistimulation with or without application of auxin (5 mM IAA or 0.1 Mm NAA). Error bars indicate SEM; n = 20. Means with different letters are significantly different (P < 0.05; ANOVA).

Figure S8

(a)

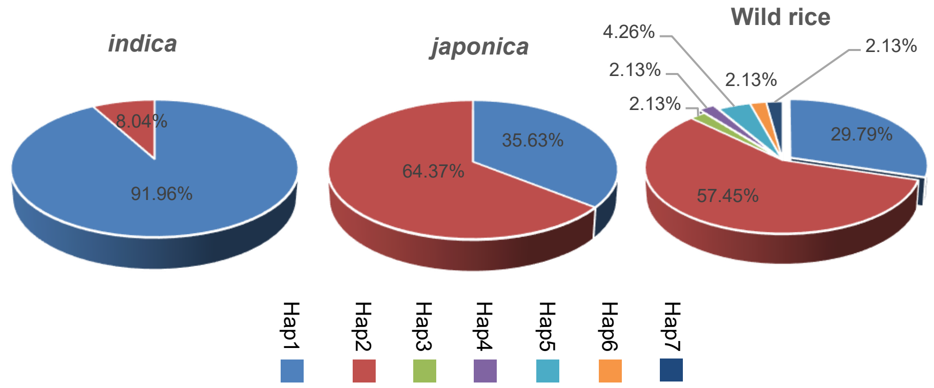

(b)

Position	368	454	832	871	1026	1051	1569	1793	No. of *indica*	No. of *japonica*	No. of Wild rice
**A.A. sub.**	**Q123L**	**S154T**	**L278V**	**N291H**	**D342E**	**L351V**	**E523D**	**I598T**			
Hap1	A	T	C	A	C	C	G	T	103	31	12
Hap2	A	T	C	A	C	C	G	C	9	56	22
Hap3	A	A	C	A	C	C	G	T	0	0	1
Hap4	A	T	C	A	A	C	G	T	0	0	1
Hap5	A	T	C	A	C	C	C	T	0	0	2
Hap6	A	T	G	C	C	G	G	T	0	0	1
Hap7	T	T	C	A	C	C	G	C	0	0	1

Figure S8. *TAC4* variants in wild and cultivated rice.

(a) Haplotype frequencies are shown as percentages for a core collection of wild and cultivated rice germplasms.

(b) The polymorphic nucleotides of Hap1–7 in the *TAC4* coding sequence

Figure S9

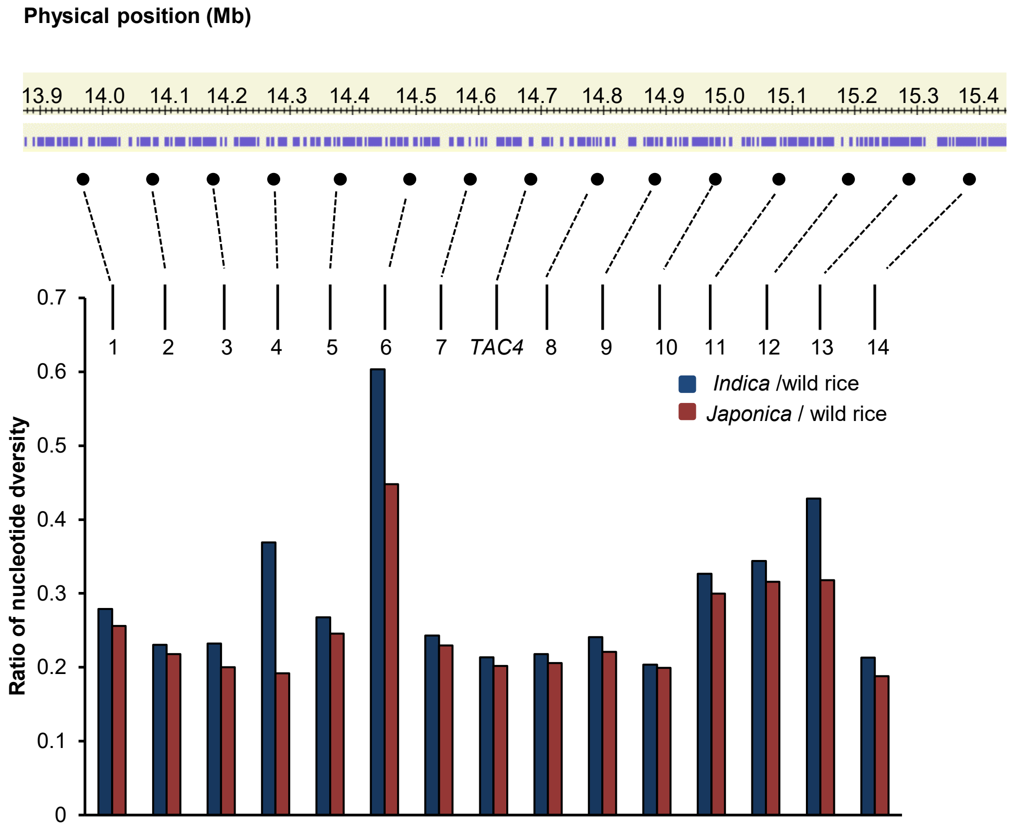

Figure S9. Comparison of diversity ratios surrounding the *TAC4 locus.*

Figure S10

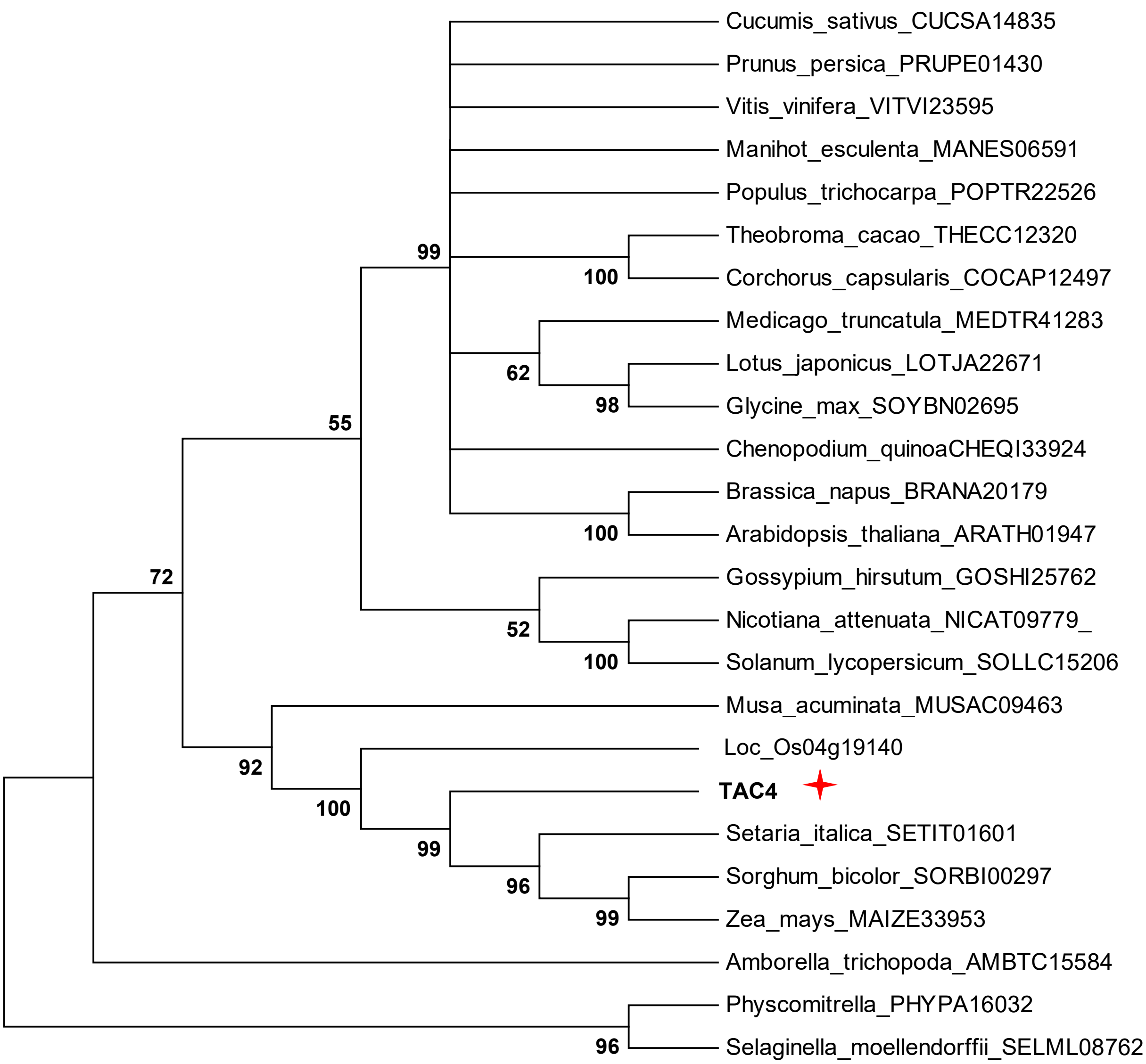

Figure S10. Phylogenetic tree of 25 homologs of TAC4 in land plants. The tree was constructed using MEGA 6.0 based on the neighbor joining method. The red asterisks indicates the position of TAC4.

Figure S11

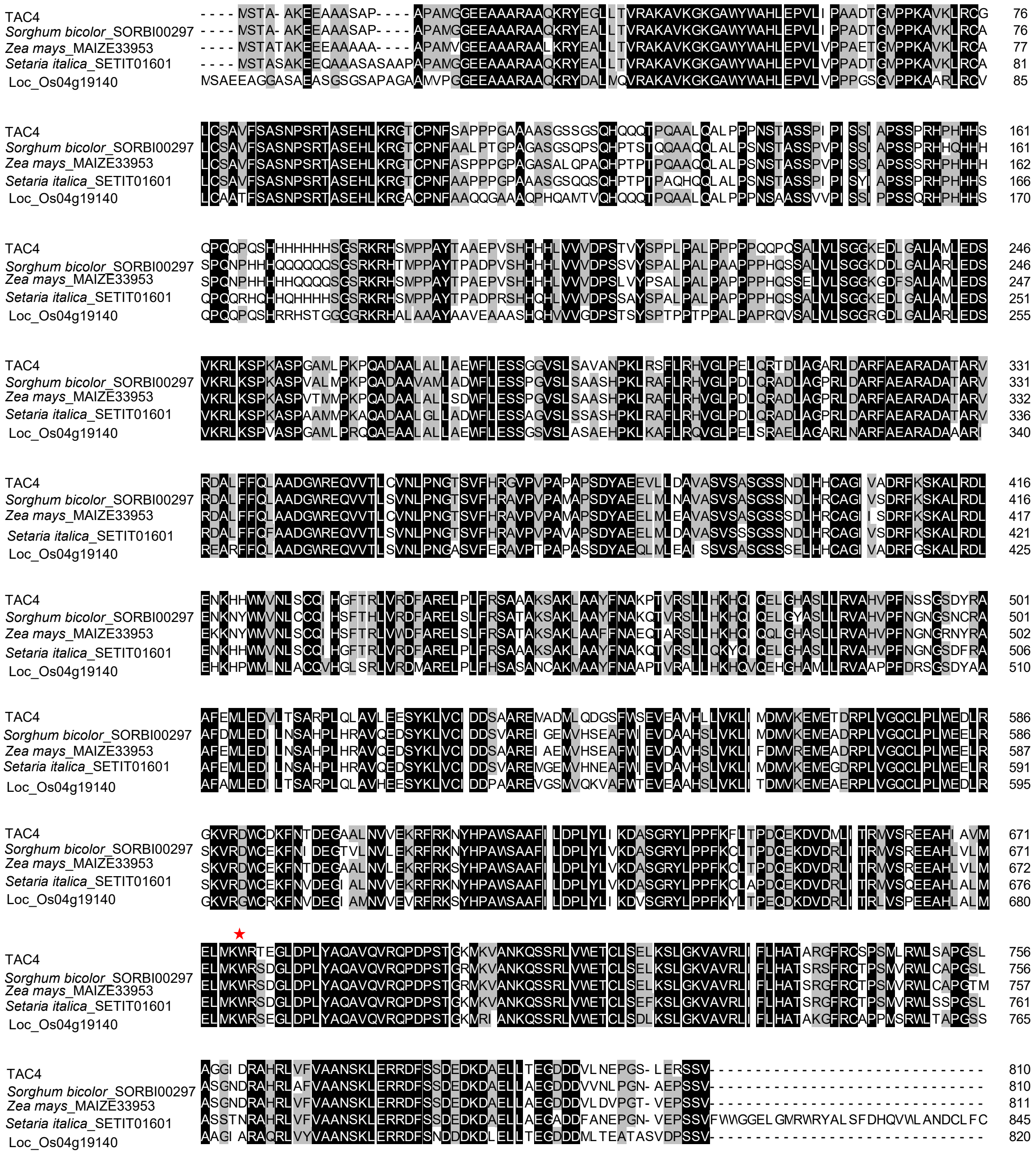

Figure S11. Multiple alignment of TAC4 and its homologs. Conserved amino acids are highlighted in shades of black and gray: white letters with black background (100% identity), black letters with gray background (80% identity). The red asterisks indicates the position of tac4 stop codon.

Figure S12

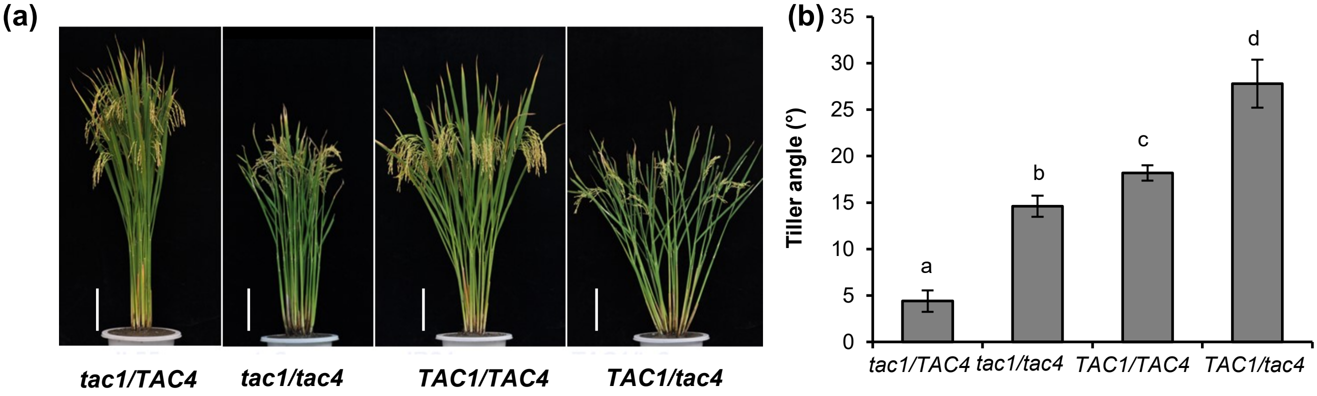

Figure S12. ***TAC4*****’s regulation of tiller angle is independent of** ***TAC1.***

(a) The morphology of *tac1/TAC4* (IL55), *tac1/tac4*, *TAC1/TAC4*, and *TAC1/tac4*. Scale bar, 20 cm.

(b) Comparison of tiller angle. Error bars indicate SEM, n = 5. Means with different letters are significantly different ($P < 0.05$; ANOVA).
