# Supplementary material for: TAC4 controls tiller angle by regulating the endogenous auxin content and distribution in rice
Source: Plant Biotechnol J. 2020 Jul 20;19(1):64–73. doi: 10.1111/pbi.13440 (PMC7769243; doi:10.1111/pbi.13440)
Supplement: Supplementary file 2 — Table S1 Comparison of agronomic traits of IL55 and tac4 plants. Table S2 Summary of the TAC4 allele type in each wild strain and cultivar in the rice germplasm core collection. Table S3 Nucleotide diversity and Tajima’s D test. Table S4 The Fst analysis at the TAC4 locus. Table S5 Primers used in the study. [file PBI-19-64-s001.pdf]

Table S1. Comparison of agronomic traits of IL55 and *tac4* plants.

traits	SH55	*tac4*
Tiller number	11.80±1.65	13.44±2.08
Panicle length (cm)	23.46±0.56	15.65±0.23**
Primary branch	12.10±0.26	10.83±0.71**
Secondary branch	30.7±2.36	20.55±3.61**
Grain number	161.20±8.11	135.85±9.12**
seed set rates (%)	0.85±0.02	0.61±0.01**
Grain yield per plant (g)	24.21±1.59	14.05±1.74**

All phenotypic data were measured from plants grown in a paddy field under natural conditions in Beijing, China. Values are means± SD (n = 30). Student's *t*- tests were used to generate *P* values (** $P < 0.01$).

Table S2. Summary of the *TAC4* allele type in each wild strain and cultivar in the rice germplasm core collection.

Variety	species	Subspecies	Origin	allele type
Nanxiongzaoyouzhan	*O. sativa*	*indica*	China	Hap1
Xiangaizao 10	*O. sativa*	*indica*	China	Hap1
Nantehao	*O. sativa*	*indica*	China	Hap1
Zaoxian140	*O. sativa*	*indica*	China	Hap1
Taishannuo	*O. sativa*	*indica*	China	Hap1
Hengxianliangchunbengu	*O. sativa*	*indica*	China	Hap1
Liuyenian	*O. sativa*	*indica*	China	Hap1
Xuanenchangtanchunnian	*O. sativa*	*indica*	China	Hap1
Zaomadao	*O. sativa*	*indica*	China	Hap1
Meihuanuo	*O. sativa*	*indica*	China	Hap1
ZhuzhenB	*O. sativa*	*indica*	China	Hap1
Chaoyang1B	*O. sativa*	*indica*	China	Hap1
L310B	*O. sativa*	*indica*	China	Hap1
Jinnante43B	*O. sativa*	*indica*	China	Hap1
Zhenshan97B	*O. sativa*	*indica*	China	Hap1
Biaoxie123B	*O. sativa*	*indica*	China	Hap1
80B	*O. sativa*	*indica*	China	Hap1
Baoxie7B	*O. sativa*	*indica*	China	Hap1
Guangluai4	*O. sativa*	*indica*	China	Hap1
Aijiaonante	*O. sativa*	*indica*	China	Hap1
Shufeng101	*O. sativa*	*indica*	China	Hap1
Leihuozhan	*O. sativa*	*indica*	China	Hap1
Dongtingwanxian	*O. sativa*	*indica*	China	Hap1
Aijiaozao	*O. sativa*	*indica*	China	Hap1
Wanlixian	*O. sativa*	*indica*	China	Hap1
IR 661-1	*O. sativa*	*indica*	China	Hap1
Liusha1	*O. sativa*	*indica*	China	Hap1
Erjiunan1	*O. sativa*	*indica*	China	Hap1
Nanjing11	*O. sativa*	*indica*	China	Hap1
Chengduai3	*O. sativa*	*indica*	China	Hap1
Sanbaili	*O. sativa*	*indica*	China	Hap1
Esiniu	*O. sativa*	*indica*	China	Hap1
Heidu4	*O. sativa*	*indica*	China	Hap1
Dongtingwanxian	*O. sativa*	*indica*	China	Hap1
Xiangdao	*O. sativa*	*indica*	China	Hap1
Mamagu	*O. sativa*	*indica*	China	Hap1
Honggu	*O. sativa*	*indica*	China	Hap1
Fanhaopi	*O. sativa*	*indica*	China	Hap1
XiangaiB	*O. sativa*	*indica*	China	Hap1
Jiangnongzao1B	*O. sativa*	*indica*	China	Hap1

Dianrui409B	*O. sativa*	*indica*	China	Hap1
88B	*O. sativa*	*indica*	China	Hap1
Gu154	*O. sativa*	*indica*	China	Hap1
Gui630	*O. sativa*	*indica*	China	Hap1
Teqingxuanhui	*O. sativa*	*indica*	China	Hap1
Xianghui91269	*O. sativa*	*indica*	China	Hap1
Aimakang	*O. sativa*	*indica*	China	Hap1
Guichao2	*O. sativa*	*indica*	China	Hap1
Xiangwanxian1	*O. sativa*	*indica*	China	Hap1
Aituogu151	*O. sativa*	*indica*	China	Hap1
Huangsiguizhan	*O. sativa*	*indica*	China	Hap1
Xiangwanxian3	*O. sativa*	*indica*	China	Hap1
Chengnongshuijing	*O. sativa*	*indica*	China	Hap1
Qitougu	*O. sativa*	*indica*	China	Hap1
Jinzhinuo	*O. sativa*	*indica*	China	Hap1
Ximaxian	*O. sativa*	*indica*	China	Hap1
Wuzuihonggu	*O. sativa*	*indica*	China	Hap1
Maweinian	*O. sativa*	*indica*	China	Hap1
Taizhong65	*O. sativa*	*indica*	China	Hap1
Qingsiai16B	*O. sativa*	*indica*	China	Hap1
Yangdao2	*O. sativa*	*indica*	China	Hap1
Dianxian232	*O. sativa*	*indica*	China	Hap1
Aimi	*O. sativa*	*indica*	China	Hap1
Biwusheng	*O. sativa*	*indica*	China	Hap1
Achar Bhog	*O. sativa*	*indica*	Bangladesh	Hap1
Bhat Mukhu	*O. sativa*	*indica*	Bangladesh	Hap1
Colombo	*O. sativa*	*indica*	India	Hap1
DoDo	*O. sativa*	*indica*	Philippines	Hap1
Hr33	*O. sativa*	*indica*	Philippines	Hap1
R2389	*O. sativa*	*indica*	Philippines	Hap1
Aclt	*O. sativa*	*indica*	Philippines	Hap1
Lchsannde	*O. sativa*	*indica*	Philippines	Hap1
Rikn-2	*O. sativa*	*indica*	Philippines	Hap1
Agbede	*O. sativa*	*indica*	Philippines	Hap1
Hoshikalmi	*O. sativa*	*indica*	Philippines	Hap1
DM36	*O. sativa*	*indica*	Vietnam	Hap1
Lili356	*O. sativa*	*indica*	Korea	Hap1
NR10045-20-3-2	*O. sativa*	*indica*	Nepal	Hap1
O-Goo-Thou	*O. sativa*	*indica*	Nepal	Hap1
Mab Suri	*O. sativa*	*indica*	Malaysia	Hap1
Sein Ta lay	*O. sativa*	*indica*	Myanmar	Hap1
Horomamawee	*O. sativa*	*indica*	Sri Lanka	Hap1
Tcanuto	*O. sativa*	*indica*	Philippines	Hap1
Naw Sadui	*O. sativa*	*indica*	Thailand	Hap1

Kalu Murunga	*O. sativa*	*indica*	Pakistan	Hap1
XX-4	*O. sativa*	*indica*	Thailand	Hap1
Lateefy	*O. sativa*	*indica*	Pakistan	Hap1
Re Them Ha Dong	*O. sativa*	*indica*	Vietnam	Hap1
Sai Duong Thamhheai	*O. sativa*	*indica*	Vietnam	Hap1
Tep1	*O. sativa*	*indica*	Vietnam	Hap1
Tep2	*O. sativa*	*indica*	Vietnam	Hap1
DR32	*O. sativa*	*indica*	Vietnam	Hap1
Chakula	*O. sativa*	*indica*	Bangladesh	Hap1
Kangpur	*O. sativa*	*indica*	Bangladesh	Hap1
GH 319	*O. sativa*	*indica*	Indonesia	Hap1
Beok Montor	*O. sativa*	*indica*	Indonesia	Hap1
Bali Ketumbaran	*O. sativa*	*indica*	Indonesia	Hap1
Beri	*O. sativa*	*indica*	Bangladesh	Hap1
OKamuna	*O. sativa*	*indica*	India	Hap1
OKabahulo	*O. sativa*	*indica*	Bangladesh	Hap1
Kele	*O. sativa*	*indica*	Bangladesh	Hap1
Hongwan1	*O. sativa*	*indica*	China	Hap1
Huke3	*O. sativa*	*indica*	China	Hap1
Tieganwu	*O. sativa*	*japonica*	China	Hap1
Muguanuo	*O. sativa*	*japonica*	China	Hap1
Qitoubaigu	*O. sativa*	*japonica*	China	Hap1
AnnongwangengB	*O. sativa*	*japonica*	China	Hap1
Ninghui21	*O. sativa*	*japonica*	China	Hap1
Jan-76	*O. sativa*	*japonica*	China	Hap1
Guihuahuang	*O. sativa*	*japonica*	China	Hap1
Xiugunuo	*O. sativa*	*japonica*	China	Hap1
jixuenuo	*O. sativa*	*japonica*	China	Hap1
Jing7623	*O. sativa*	*japonica*	China	Hap1
Gongju73	*O. sativa*	*japonica*	China	Hap1
Youmangzaogeng	*O. sativa*	*japonica*	China	Hap1
Bawangbian	*O. sativa*	*japonica*	China	Hap1
Xibainian	*O. sativa*	*japonica*	China	Hap1
Xianggu	*O. sativa*	*japonica*	China	Hap1
Beizinuo	*O. sativa*	*japonica*	China	Hap1
JinghuB	*O. sativa*	*japonica*	China	Hap1
Gzhenshan97B	*O. sativa*	*japonica*	China	Hap1
Dangyu5	*O. sativa*	*japonica*	China	Hap1
Xiaohonggu	*O. sativa*	*japonica*	China	Hap1
Banjiemang	*O. sativa*	*japonica*	China	Hap1
PeiC122	*O. sativa*	*japonica*	China	Hap1
OKWR 221	*O. sativa*	*japonica*	China	Hap1
OKabonl	*O. sativa*	*japonica*	Bangladesh	Hap1
Padi Lasat	*O. sativa*	*japonica*	Malaysia	Hap1

Kasinkan	*O. sativa*	*japonica*	Japan	Hap1
Fushan	*O. sativa*	*japonica*	Japan	Hap1
Muqiuxiu	*O. sativa*	*japonica*	China	Hap1
C418	*O. sativa*	*japonica*	China	Hap1
Zegu	*O. sativa*	*japonica*	China	Hap1
Babaili	*O. sativa*	*japonica*	China	Hap1
Chikenuo	*O. sativa*	*indica*	China	Hap2
Chiem Bua	*O. sativa*	*indica*	Vietnam	Hap2
Ase Bolon Kamandi	*O. sativa*	*indica*	Indonesia	Hap2
Kathemax	*O. sativa*	*indica*	Indonesia	Hap2
Barkat	*O. sativa*	*indica*	India	Hap2
Kashmin Basmali	*O. sativa*	*indica*	Pakistan	Hap2
Basmati Dhan	*O. sativa*	*indica*	Pakistan	Hap2
Basmati Kamon	*O. sativa*	*indica*	Pakistan	Hap2
Basmati Hunar	*O. sativa*	*indica*	Pakistan	Hap2
Shuiyuan300li	*O. sativa*	*japonica*	China	Hap2
Zhonglou1	*O. sativa*	*japonica*	China	Hap2
Weiguo	*O. sativa*	*japonica*	China	Hap2
Zhonghua8	*O. sativa*	*japonica*	China	Hap2
Jindao1	*O. sativa*	*japonica*	China	Hap2
Baigedao	*O. sativa*	*japonica*	China	Hap2
Liaojing287	*O. sativa*	*japonica*	China	Hap2
Gaoyangdiandaodahongmang	*O. sativa*	*japonica*	China	Hap2
Muxiqiu	*O. sativa*	*japonica*	China	Hap2
Feidongtangdao	*O. sativa*	*japonica*	China	Hap2
Hongqi5	*O. sativa*	*japonica*	China	Hap2
Guangkexiangnuo	*O. sativa*	*japonica*	China	Hap2
Libu	*O. sativa*	*japonica*	Philippines	Hap2
Kutube	*O. sativa*	*japonica*	Philippines	Hap2
Lepqu	*O. sativa*	*japonica*	Philippines	Hap2
Mack Kouk	*O. sativa*	*japonica*	Laos	Hap2
Rasi Pagalou	*O. sativa*	*japonica*	Malaysia	Hap2
Chabli Chabol Glang	*O. sativa*	*japonica*	Thailand	Hap2
Do Khao	*O. sativa*	*japonica*	Laos	Hap2
Lepsang	*O. sativa*	*japonica*	Laos	Hap2
Agami M1	*O. sativa*	*japonica*	Philippines	Hap2
Lukip	*O. sativa*	*japonica*	Philippines	Hap2
Xitong400	*O. sativa*	*japonica*	Korea	Hap2
Qingfeng	*O. sativa*	*japonica*	Japan	Hap2
Dupanyui	*O. sativa*	*japonica*	Indonesia	Hap2
Heritam	*O. sativa*	*japonica*	Indonesia	Hap2
Daoli	*O. sativa*	*japonica*	Japan	Hap2
Huanghai33	*O. sativa*	*japonica*	Korea	Hap2
Duangennuo	*O. sativa*	*japonica*	Japan	Hap2

Pingbei5	*O. sativa*	*japonica*	Korea	Hap2
Pingbei9	*O. sativa*	*japonica*	Korea	Hap2
Milizu	*O. sativa*	*japonica*	Korea	Hap2
Xinchuanchinuo	*O. sativa*	*japonica*	Korea	Hap2
Xiaoluohan	*O. sativa*	*japonica*	China	Hap2
Changzhong	*O. sativa*	*japonica*	China	Hap2
Sanchaoqi	*O. sativa*	*japonica*	China	Hap2
Huangdao	*O. sativa*	*japonica*	China	Hap2
Putaolvzhong	*O. sativa*	*japonica*	China	Hap2
Jinlaotou	*O. sativa*	*japonica*	China	Hap2
Sanyiqian	*O. sativa*	*japonica*	China	Hap2
Chenjianuo	*O. sativa*	*japonica*	China	Hap2
Cunsanli	*O. sativa*	*japonica*	China	Hap2
Ganshangchen	*O. sativa*	*japonica*	China	Hap2
PADI PENOKULU	*O. sativa*	*japonica*	Indonesia	Hap2
PADI KEPOK	*O. sativa*	*japonica*	Indonesia	Hap2
Taibei309	*O. sativa*	*japonica*	Chinese Taipei	Hap2
LUK TAKHAA	*O. sativa*	*japonica*	Afghanistan	Hap2
Laohuzhong	*O. sativa*	*japonica*	China	Hap2
Xingguo	*O. sativa*	*japonica*	China	Hap2
Baimaodao	*O. sativa*	*japonica*	China	Hap2
Longhuamaohulu	*O. sativa*	*japonica*	China	Hap2
Nantiangangjiugu	*O. sativa*	*japonica*	China	Hap2
Mubanggu	*O. sativa*	*japonica*	China	Hap2
Methi Noi	*O. sativa*	*japonica*	Laos	Hap2
Pingrang10	*O. sativa*	*japonica*	Korea	Hap2
Gaoliqiu	*O. sativa*	*japonica*	Korea	Hap2
ruf01	*O. rufipogon*	perennial	Philippines	Hap1
ruf02	*O. rufipogon*	perennial	Thailand	Hap1
ruf03	*O. rufipogon*	perennial	Bangladesh	Hap1
ruf04	*O. rufipogon*	perennial	China	Hap2
ruf05	*O. rufipogon*	perennial	China	Hap2
ruf06	*O. rufipogon*	perennial	China	Hap2
ruf07	*O. rufipogon*	perennial	China	Hap2
ruf08	*O. rufipogon*	perennial	China	Hap2
ruf09	*O. rufipogon*	perennial	China	Hap2
ruf10	*O. rufipogon*	perennial	China	Hap2
ruf11	*O. rufipogon*	perennial	China	Hap2
ruf12	*O. rufipogon*	perennial	china	Hap2
ruf13	*O. nivara*	annual	Chinese Taipei (Taiwan)	Hap2
ruf14	*O. rufipogon*	perennial	China	Hap2
ruf15	*O. rufipogon*	perennial	China	Hap2
ruf16	*O. rufipogon*	perennial	China	Hap2

ruf17	*O. rufipogon*	perennial	China	Hap2
ruf18	*O. rufipogon*	perennial	China	Hap2
ruf19	*O. rufipogon*	perennial	China	Hap2
ruf20	*O. rufipogon*	perennial	China	Hap2
ruf21	*O. rufipogon*	perennial	China	Hap2
ruf22	*O. rufipogon*	perennial	China	Hap2
ruf23	*O. rufipogon*	perennial	China	Hap2
ruf24	*O. rufipogon*	perennial	Cambodia	Hap1
ruf25	*O. rufipogon*	perennial	Nepal	Hap1
ruf26	*O. rufipogon*	perennial	Bangladesh	Hap1
ruf27	*O. rufipogon*	perennial	Philippines	Hap1
ruf28	*O. rufipogon*	perennial	Laos	Hap1
ruf29	*O. nivara*	perennial	India	Hap1
ruf30	*O.rufipogon*	perennial	China	HAP2
ruf31	*O.rufipogon*	perennial	India	Hap2
ruf32	*O.rufipogon*	perennial	China	Hap5
ruf33	*O.rufipogon*	perennial	China	Hap5
ruf34	*O.nivara*	annual	Laos	Hap1
ruf35	*O.nivara*	annual	Sri Lanka	Hap1
ruf36	*O.rufipogon*	perennial	India	Hap3
ruf37	*O.rufipogon*	perennial	Bangladesh	Hap4
ruf38	*O.rufipogon*	perennial	China	Hap6
ruf39	*O.rufipogon*	perennial	Costarica	Hap7
ruf40	*O.rufipogon*	perennial	China	Hap1

Table S3. Nucleotide diversity and Tajima's *D* test.

	N	L	S	H	π	θw	Tajima' *s D*
O. sativa	199	2433	3	3	0.00052	0.00021	2.3869
indica	112	2433	3	3	0.00015	0.00023	-0.6179
Japonica	87	2433	3	3	0.00059	0.00024	2.5751*
wild rice	40	2433	26	14	0.00114	0.00251	-1.8554*

N, total number of sequences; L, average length (bp) of the sequences per taxon; S, number of polymorphic (segregating) sites; H, number of haplotypes; π, average number of pairwise nucleotide differences per site calculated based on the total number of polymorphic sites; θw, Watterson's estimator of per base pair calculated based on the total number of polymorphic sites. Student's *t* tests were used to generate *P* values (* $P < 0.05$).

Table S4. The F_{st} analysis at the *TAC4* locus.

Population 1	Population 2	F_{st}
ind	*jap*	0.49803
ind	cultivates	0.16950
ind	wild	0.23977
jap	cultivates	0.16867
jap	wild	0.28847
cultivates	wild	0.17564

Table S5. Primers used in the study.

Name	Forward primers（5'-3'）	Reverse primers（5'-3'）	Purpose
RM555	TTGGATCAGCCAAAGGAGAC	CAGCATTGTGGCATGGATAC	mapping
RM71	CTAGAGGCGAAAACGAGATG	GGGTGGGCGAGGTAATAATG	mapping
RM7632	TATTTCCTCGCCTATCTGCG	AAAGGTTGAGCACATGGACC	mapping
RM3688	GTTGAATCAAGCTGTGCAGC	AGCTAGGCAAAGCATGCATG	mapping
RM3874	TGGGTGATCTTAGTTTGGCC	AATGTGCCTGCACATGTCAC	mapping
RM13071	ATGTGGGTGACGTACGGAACTTTG	GGTAGGATTCCTTCGGATAGACAG	mapping
ID13046	TTAGAAGGGAGTAAGGGTGTC	TATTAAAATCGCTCGTATTAATG	mapping
ID15467	GAACAAACGCAGAGCACCTC	TTGCATTAGCGGTTATTACTAGC	mapping
ID16147	GCTGTTGGGCATTCATTTAT	CTGTTGTCTCCCGTATAGATGTG	mapping
TAC4	AAGGTCGCAAACAAGCAG	AACTGCCAGGAGCAGAGAG	qRT-PCR
OsIAA20	TGGCGGATATGTGAAGGTGAA	TATGAGCCGAGGATGGACAAG	qRT-PCR
YUCCA1	TCATCGGACGCCCTCAACGTCGC	GGCAGAGCAAGATTATCAGTC	qRT-PCR
YUCCA2	GTCCAAAGGGAGGAGTCGTCCAG	GCATGATGTTTACACCCGGCCTT	qRT-PCR
YUCCA3	GTGAGAACGGGCTCTACTCGGTC	GCTTATGCATGACCGATGAACAC	qRT-PCR
YUCCA4	GCAGAATGGCCTGTACGCTGTTG	CAGACCAGCACATGACGTGTCTA	qRT-PCR
YUCCA5	CCTCCTACGACGCCGCCATGATC	CTCCCAACACAGCGACGACAGAA	qRT-PCR
YUCCA6	CCATTCCCAGATGGTTGGAAGG	CATGTTGCGCCTCAAGATATTTG	qRT-PCR
YUCCA7	CACTGCTGTGTCCTACAATATCAC	GGAGGTGCATCTCCGTCATCTTC	qRT-PCR
TAR1	GTGGGCGCTGGTGAAGG	CGGGCGAAGTGGAACAGTC	qRT-PCR
TAR2	CCACTCCTCTTCGCCTGTTC	CACATCCTCCCTATCGCACC	qRT-PCR
TAC1	GAGATGGCTCTAAAGGTGTTC	CGTGCCAATTGCAAGTATACC	qRT-PCR
Actin	TACAGTGTCTGGATTGGAGGAT	TCTTGGCTTAGCATTCTTGGGT	qRT-PCR
TAC4-OE	CGCGGTACCAGGAGGTTCTCTCTTGT GGTC	GGACTAGTAGAGTGAGGAAGTAAGAA TTAACAG	overexpression
TAC4-RNAi	GGACTAGTGTGGCGAACCCGAAACT	TTCGAGCTCGAAGCCATGGATTTGGC A	TAC4-RNAi
TAC4-2	CGCGGTACCAGGAGGTTCTCTCTTGT GGTC	GCTCTAGACACTGAGGAGCGCTCCA	TAC4-GFP
pTAC4	CGCGGATCCTGTAGCGTATGAGAGCA TGTGAA	TGCACTGCAGTGGCGGCCGTCGACA T	*pTAC4:GUS*
